# Direct susceptibility testing by disk diffusion on clinical samples: a rapid and accurate tool for antibiotic stewardship

**DOI:** 10.1007/s10096-015-2349-2

**Published:** 2015-02-20

**Authors:** L. Coorevits, J. Boelens, G. Claeys

**Affiliations:** Ghent University, Ghent, Belgium

## Abstract

We compared the accuracy of direct susceptibility testing (DST) with conventional antimicrobial susceptibility testing (AST), both using disk diffusion, on clinical samples. A total of 123 clinical samples (respiratory tract samples, urine, vaginal and abdominal abscess discharges, bile fluid and a haematoma punctate) were selected on various indications; direct inoculation on Mueller–Hinton agar and antibiotic paper disks were applied. In parallel, standard culture, identification and AST on the colonies grown overnight was executed. Both AST and DST were interpreted after identification of the isolates. The results from both AST and DST for 11 antibiotics tested on 97 samples with Gram-negative rods showed 93.4 % total agreement, 1.6 % minor discordances, 4.6 % major discordances and 0.4 % very major discordances. Analysing the discordant results, DST predominantly resulted in more resistant isolates than AST. This was mostly due to the presence of resistant mutants or an additional isolate. The remaining discordances were seen for isolates with inhibition zones close to the clinical breakpoint. For the 26 samples yielding staphylococci, a total agreement of 100 % was observed for the nine antibiotics tested. Overall, the highest percentage of discordant results occurred for the β-lactam antibiotics amoxicillin–clavulanate (13.4 %) and cefuroxime (12.4 %). When used selectively and interpreted carefully, DST on clinical samples is potentially very useful in the management of critically ill patients, as the time to results is shortened by approximately 24 h. However, we recommend to communicate results with reservations and confirm by conventional AST.

## Introduction

The identification of clinically significant bacteria in the laboratory and the performance of antimicrobial susceptibility testing (AST) provides information essential for an accurate management of patients with a bacterial infection [1]. However, results are only available with a delay of 48–72 h after sampling, as bacteria need to be cultured before AST can be executed. Meanwhile, the patients must receive empirical antibiotherapy. The diminishing and unpredictable susceptibility to antibiotic agents can lead to inadequate therapy and urges the empiric use of broad-spectrum antibiotics. De-escalation of treatment is practised only when results from AST are available, with immediate and long-term consequences such as the emergence of multidrug-resistant micro-organisms and an increased risk of severe superinfections, morbidity, mortality and costs [2].

The most commonly used methods for AST are conventional phenotypic methods, based on culturing on agar (e.g. disk diffusion tests) or on microtitration plates (e.g. broth dilution tests) [3]. Disk diffusion has many advantages, as it is cheap, flexible and allows visibility of growth, correct inoculum, mixed cultures and other abnormalities. Another benefit is the possibility of executing direct susceptibility testing (DST). Aiming at a shortened turnaround time, DST has been practised in some laboratories and reported in multiple papers [2–21]. Nevertheless, the American Society for Microbiology (ASM) [22], the British Society for Antimicrobial Chemotherapy (BSAC) [23] and the European Committee on Antimicrobial Susceptibility Testing (EUCAST) [24] seriously criticise DST, since the inoculum is not standardised. On the other hand, providing clinicians with early microbiological information has a beneficial impact on the patient, permitting tailored antibiotic use and a decrease in antimicrobial-related adverse events [25]. Besides, previous experiments executed in our laboratory on the comparability between AST and DST in respiratory samples showed promising results (unpublished data). The aim of our study was to compare DST on clinical samples with conventional AST, both by disk diffusion, after the recent introduction of rapid identification by matrix-assisted laser desorption ionisation time-of-flight mass spectrometry (MALDI-TOF MS).

## Materials and methods

### Specimen collection

A total of 123 clinical samples were selected on various indications, such as request by the clinician for DST, a new infection in a critically ill patient or a Gram stain showing predominantly staphylococci or Gram-negative rods (GNR). The studied specimens were 48 endotracheal aspirates, 13 sputum samples, three bronchoalveolar lavages, 28 catheterised and 21 midstream urine specimens, two nephrostomy aspirates, discharges from three vaginal to three abdominal abscesses, one bile fluid and one haematoma punctate.

### Culture

Urine samples were cultured on tryptic soy agar with 5 % sheep blood (Becton Dickinson Diagnostic Systems, Sparks, MD, USA) and MacConkey agar (Becton Dickinson Diagnostic Systems, Sparks, MD, USA). Respiratory tract samples were cultured on Columbia agar with 5 % sheep blood (Becton Dickinson Diagnostic Systems, Sparks, MD, USA), MacConkey agar, tryptic soy agar with 5 % sheep blood and *Haemophilus* chocolate 2 agar (bioMérieux, La Balme-les-Grottes, France). The other samples were cultured on MacConkey agar, mannitol salt agar (Becton Dickinson Diagnostic Systems, Sparks, MD, USA) and tryptic soy agar with 5 % sheep blood. The isolates were identified with MALDI-TOF MS (Bruker Daltonik GmbH, Bremen, Germany).

### Antimicrobial susceptibility testing

Disk diffusion AST and DST was performed using paper disks (Bio-Rad Laboratories, Hercules, CA, USA) on Mueller–Hinton agar (Bio-Rad Laboratories, Hercules, CA, USA). For GNR, 13 antibiotics were tested: ampicillin (AMP), amoxicillin–clavulanate (AMC), piperacillin–tazobactam (PPT), temocillin (TEM), meropenem (MEM), cefuroxime (CXM), cefotaxime (CTX), ceftazidime (CAZ), gentamicin (GEN), amikacin (AMI), ofloxacin (OFL), nitrofurantoin (FUR) and trimethoprim–sulfamethoxazole (SXT). For staphylococci, nine antibiotics were tested: penicillin (PEN), cefoxitin (FOX), erythromycin (ERY), clindamycin (CLI), linezolid (LIN), gentamicin (GEN), rifampicin (RIF), nitrofurantoin (FUR) and trimethoprim–sulfamethoxazole (SXT). Zones of inhibition were interpreted as susceptible (S), intermediate susceptible (I) or resistant ®) according to the EUCAST guidelines [26] for all antibiotics, except for temocillin [27]. For DST, a sterile cotton swab was dipped into a vortexed sample and inoculated onto a Mueller–Hinton agar plate, following a massive three directions pattern. AST with disk diffusion was executed according to the EUCAST guidelines [26]. Both AST and DST plates were read simultaneously after overnight incubation at 35 °C, together with the identification of the isolates using species-specific breakpoints. All results were interpreted by double-blind observations by two experienced lab technicians. When two or more pathogens were isolated, the results of DST were compared with the cumulative susceptibility of the different isolates found with the regular technique. Isolates recovered with only one of two methods were marked as additional isolates. Samples with weak growth on DST were excluded, because of the unreliable readout.

## Results

### GNR

From 42 urinary tract samples yielding GNR, 28 were monomicrobial and 14 were polymicrobial; 24 *Escherichia coli*, 11 *Proteus mirabilis*, nine *Klebsiella pneumoniae*, four *Pseudomonas aeruginosa*, three *Morganella morganii*, three *Enterobacter cloacae*, one *Acinetobacter baumannii*, one *Proteus vulgaris*, one *Klebsiella oxytoca* and one *Proteus penneri*. From the 47 respiratory tract samples positive with GNR, 28 were monomicrobial and 19 were polymicrobial; 18 *P. aeruginosa*, 13 *E. coli*, six *K. pneumoniae*, six *Serratia marcescens*, four *M. morganii*, three *E. cloacae*, three *Enterobacter aerogenes*, three *P. mirabilis*, two *Serratia liquefaciens*, two *S. maltophilia*, one *Acinetobacter* spp., one *Burkholderia cepacia*, one *Citrobacter braakii*, one *Citrobacter* spp., one *K. oxytoca*, one *Proteus* spp. and one *Pseudomonas* spp. strains. From the three vaginal specimens with GNR, two were monomicrobial and one was polymicrobial; three *K. pneumoniae* and one *E. coli*. From the three abdominal specimens with GNR, two were monomicrobial and one was polymicrobial; three *E. coli* and one *K. pneumoniae*. The bile fluid specimen yielded four different GNR: *E. coli*, *K. oxytoca*, *E. aerogenes* and *P. aeruginosa*. The haematoma punctate was positive for *E. coli*.

AST and DST from 97 clinical samples yielded 1,261 antibiotic–isolate combinations. The results obtained with both techniques were in overall agreement in 93.4 % (1,178/1,261) of cases. The remaining comparisons showed a minor discordance (S vs. I or I vs. R) in 1.6 % (20/1,261), a major discordance (S in AST vs. R in DST) in 4.6 % (58/1,261) and a very major discordance (R in AST vs. S in DST) in 0.4 % (5/1,261) of cases (Table 1). The discordant cases in which DST yielded more resistant results than AST (major discordances) are presented in lines 1 to 3 of Table 2. 87 % of those discrepancies are explained by the recovery of resistant mutants or the presence of an additional isolate not found in the regular processing of the sample. For all other discrepancies, inhibition zone diameters were close to the clinical breakpoint, where small differences (≤3 mm) yield a different SIR result. This phenomenon also accounts for all of the cases where AST shows more resistant results than DST (very major discordances), presented in lines 4 to 6 of Table 2.

**Table 1:** Comparison of antimicrobial susceptibility testing (AST) and direct susceptibility testing (DST) of the 1,261 antibiotic–isolate samples yielding Gram-negative rods (GNR)

		AST		
		S	I	R
DST	S	761 (60.3 %)	2^a^ (0.2 %)	5^c^ (0.4 %)
	I	8^a^ (0.6 %)	14 (1.1 %)	2^a^ (0.2 %)
	R	58^b^ (4.6 %)	8^a^ (0.6 %)	403 (32.0 %)

^a^Minor discordances

^b^Major discordances

^c^Very major discordances

**Table 2:** Explanation of the discordant results for GNR

		RM	AI	BP	Total
1	DST R vs. AST S	10	45	3	58
2	DST R vs. AST I	5	1	2	8
3	DST I vs. AST S	3	0	5	8
4	DST S vs. AST R	0	0	5	5
5	DST I vs. AST R	0	0	2	2
6	DST S vs. AST I	0	0	2	2

*RM* resistant mutant; *AI* additional isolate; *BP* inhibition zone close to breakpoint

Line 1: major discordances; lines 2, 3, 5, 6: minor discordances; line 4: very major discordances

### Staphylococci

From the 26 samples yielding staphylococci, all were monomicrobial. From 17 respiratory tract samples, we isolated 13 methicillin-susceptible *Staphylococcus aureus* (MSSA), three methicillin-resistant *S. aureus* (MRSA) and three *S. epidermidis*. From nine urinary tract samples, we isolated thre MRSA, three *S. epidermidis*, two MSSA and one *S. haemolyticus*. A total of 43 clinical isolates accounted for 234 antibiotic–isolate combinations, with an overall agreement of 100 %.

Table 3 summarises the overall agreement for each antimicrobial agent tested against GNR and/or staphylococci. The highest percentage of discordant results occurred in the β-lactam antibiotics AMC (13.4 %) and CXM (12.4 %). No differences were found for FOX, PEN, ERY, RIF, CLI, FUR and LIN.

**Table 3:** Overall agreement and reasons for discordance for each antimicrobial agent tested for GNR^a^ and/or staphylococci^b^

	Agreement		Discordance					
			RM		AI		BP	
	*n*	%	*n*	%	*n*	%	*n*	%
Total	1,410	94.3 %	18	1.2 %	48	3.2 %	19	1.3 %
AMC^a^	84	86.6 %	0	0.0 %	10	10.3 %	3	3.1 %
CXM^a^	85	87.6 %	3	3.1 %	8	8.2 %	1	1.0 %
FUR^a,b^	115	93.5 %	0	0.0 %	4	4.1 %	4	4.1 %
PPT^a^	89	91.8 %	6	6.2 %	0	0.0 %	2	2.1 %
CAZ^a^	90	92.8 %	4	4.1 %	3	3.1 %	0	0.0 %
TEM^a^	91	93.8 %	0	0.0 %	3	3.1 %	3	3.1 %
OFL^a^	91	93.8 %	1	1.0 %	4	4.1 %	1	1.0 %
AMI^a^	92	94.8 %	2	2.1 %	2	2.1 %	1	1.0 %
CTX^a^	92	94.8 %	1	1.0 %	4	4.1 %	0	0.0 %
AMP^a^	93	95.9 %	1	1.0 %	3	3.1 %	0	0.0 %
MEM^a^	93	95.9 %	0	0.0 %	1	1.0 %	3	3.1 %
SXT^a,b^	119	96.7 %	0	0.0 %	4	4.1 %	0	0.0 %
GEN^a,b^	120	97.6 %	0	0.0 %	2	2.1 %	1	1.0 %
FOX^b^	26	100.0 %	0	0.0 %	0	0.0 %	0	0.0 %
PEN^b^	26	100.0 %	0	0.0 %	0	0.0 %	0	0.0 %
ERY^b^	26	100.0 %	0	0.0 %	0	0.0 %	0	0.0 %
RIF^b^	26	100.0 %	0	0.0 %	0	0.0 %	0	0.0 %
CLI^b^	26	100.0 %	0	0.0 %	0	0.0 %	0	0.0 %
LIN^b^	26	100.0 %	0	0.0 %	0	0.0 %	0	0.0 %

*RM* resistant mutant; *AI* additional isolate; *BP* inhibition zone close to breakpoint

## Discussion

In our study, we compared both AST and DST by disk diffusion on 123 clinical samples, mainly urine and respiratory tract samples. The agreement for each individual antibiotic agent always exceeded 86 %. An important thing to notice is that, in 89 % of all discordant cases, DST showed more resistant results than AST, mainly due to the recovery of resistant mutants or an additional isolate.

Numerous studies have been published on DST on different sample types, most of them showing excellent agreement between AST and DST. All studies on urine samples comparing both AST and DST by disk diffusion show an overall agreement of over 90 % [4, 7, 11–13, 19]. Studies on urine samples comparing AST by disk diffusion with DST by other techniques (e.g. disk elution, API systems, Autobac, agar dilution, broth microdilution) had similar results [5, 6, 8–10, 14, 18]. Studies on respiratory tract specimens compared DST by the Etest with AST by disk diffusion [16, 17, 21] or with AST by broth microdilution [2, 20]. All showed an overall agreement of over 85 %, except for a study on the sputa of cystic fibrosis patients positive with *P. aeruginosa* [21], which showed a poor overall agreement, especially for aminoglycosides (60 %). One study on intra-ocular aspirations, comparing both AST and DST by disk diffusion, found an overall agreement of 88 % [15]. The poorest results were noted in a study on wound exudates, comparing both AST and DST by disk diffusion [3]. The total agreement was no more than 39 %, mainly due to inadequate growth on most of the DST plates. It should be emphasised that the authors placed the swabs, taken from the wound exudates, in 1 mL of tryptic soy broth, thereby diluting the samples.

The main advantage of DST is that it offers the possibility of obtaining results 24 h earlier than conventional AST, as has been reported in the literature [2, 12, 15]. At that time, possible pathogens can be identified from culture with the recently introduced fast identification tools, such as MALDI-TOF MS, whereby results from culture and DST can be interpreted together. Even if susceptibility testing is incomplete, a specific phenotype can appear, redirecting the empirically started antibiotic treatment. Furthermore, the use of the whole sample incorporates the physical characteristics of sample itself, including mucoid bacterial phenotypes and antibiotic penetration. Thus, the results are likely to be more clinically applicable. In addition, DST determines the antibiotic sensitivity for the whole sample, rather than individual cultured colonies, enabling an assessment of the population susceptibility to antibiotics. The proof that DST indeed looks at the bigger picture is found in our own experiments, where the recovery of resistant mutants or an additional isolate explain the majority of the discordant cases. This was particularly true for AMC and CXM, antibiotics with quite a small spectrum of activity, where AMC- and/or CXM-resistant mutants or additional isolates were frequently found. Furthermore, in our study, we included two consecutive urine samples of a patient infected with *K. pneumoniae*, of which DST of the first sample showed more resistance than AST for AMC ® vs. S) and PPT (I vs. S). In the second sample, however, AST agreed on the more resistant DST results, thereby proving the initial false susceptibility.

In polymicrobial specimens, isolates with similar phenotypic properties can be easily considered as one, especially when present in small numbers. This phenomenon is well illustrated in our study by a case of a polymicrobially infected bile fluid, where AST revealed three species (*E. coli*, *K. oxytoca* and *P. aeruginosa*), whilst DST revealed the presence of a fourth species (*E. aerogenes*) by discordance in resistance to AMC, CXM, CTX, CAZ, SXT and OFL, each R in DST and S in AST. The more resistant *E. aerogenes* was cultured eventually in a follow-up sample taken 8 days later. The clinical impact of DST was evaluated in a prospectively and randomised study [25], showing a significantly higher percentage of patients receiving an adequate defined daily dose of therapy in the DST group (91.3 %) compared to the AST group (68.3 %). Moreover, rapid identification and susceptibility testing has been shown to reduce the length of hospitalisation of patients with sepsis, which translates into reductions in the cost of patient care [22].

On the other hand, DST has several potential drawbacks, including the likelihood of a non-interpretable DST with low bacterial concentrations and ambiguities due to mixed cultures. Due to the unstandardised inoculum in DST, it is hardly surprising that strains showing susceptibility around the clinical breakpoint can easily switch between interpretation categories. This is particularly the case in antibiotics having no (AMC, CXM and FUR) or small (PPT) intermediate susceptible category. In our experiments, this phenomenon was the number two reason for discordant results. Moreover, all of the five very major discordances ® in AST vs. S in DST) showed a susceptibility close to the breakpoint (three FUR, one AMC and one CXM). The issue of the unstandardised inoculum could theoretically be circumvented by the use of Etests, which contain a predefined stable gradient and are an inoculum-tolerant system [2, 17]. However, as described above, the results from studies using the Etest are equal to those using disk diffusion. In addition, in case of co-infections, the readability of disk diffusion surpasses the Etest.

DST is not recommended by the ASM [22, 28], the BSAC [23] nor the EUCAST [24], as the inoculum is unstandardised. According to the ASM, DST of positive blood cultures and urine is acceptable under certain conditions. First, microscopy has to suggest a monomicrobial infection. Second, confirmatory AST has to be repeated once the organism is available in pure culture. Next, laboratories should validate their method against the standard results and DST results should only be read by an experienced microbiologist. The BSAC acknowledges the common practice of DST in many laboratories. They suggest that a correct inoculum is achieved for urine by using a sterile 10-μL loop and/or a sterile cotton swab for blood cultures by placing one drop of broth in 5 mL of sterile water, dependent on the results of the Gram stain. Besides, they recommend tests to be repeated in case of non- or semi-confluent growth, or mixed culture. The EUCAST stresses that there are currently no validated methods for correct inoculums, but advocate a minimal incubation time of 16 h and caution for visibly light inoculum, as strains could be reported falsely susceptible. They claim that a reliable interpretation of results requires a correct identification of the species and repeat testing on pure cultures. The EUCAST does not consider automated systems suited for DST. Moreover, in general, they comment that any laboratory using this approach must take responsibility for ensuring reliable results. In our approach, most of these considerations are taken into account, with the incorporation of rapid identification with MALDI-TOF MS. Despite not having a solution for inoculum standardisation, our results were very similar to the standard technique.

Our study has some limitations, especially as we did not systematically analyse the impact of DST on antibiotic prescription and clinical outcome. However, anecdotally, we have found DST to be very useful in antibiotic decision-making. Besides, we did not standardise the inoculum. In order to demonstrate the wide applicability of DST, we included up to six different sample types. Evidentially, not every category was equally represented, resulting in limited data of certain sample types.

In conclusion, DST on clinical samples is potentially very useful in the management of critically ill patients, as the time required for antibiotic sensitivity results is shortened by approximately 24 h. Rather than the susceptibility of one isolate, DST reflects the microbial community in the sample, with resistant mutants and additional isolates already visible. However, the results should be communicated as provisional and confirmed by conventional AST. DST can be introduced in laboratories using disk diffusion testing in a well-defined sample selection, such as the urine of patients with pyelonephritis or even community-acquired cystitis, respiratory samples in severe pneumonia, other severe infections and more general in situations, regions or institutes with high resistance rates.
